# Assessment of Adherence to Clinical Guidelines in Patients with Chronic Hepatitis B

**DOI:** 10.3390/v14102229

**Published:** 2022-10-11

**Authors:** Warshan K. Katwaroe, Sylvia M. Brakenhoff, Daniël P. C. van der Spek, Robert J. de Knegt, Laurens A. van Kleef, Robert A. de Man, Adriaan J. P. van der Meer, Milan J. Sonneveld, the ICARUS Study Group

**Affiliations:** Department of Gastroenterology and Hepatology, Erasmus MC University Medical Center, 3015 GD Rotterdam, The Netherlands

**Keywords:** treatment eligibility, treatment evaluation, management of care, HCC surveillance, medical guidelines

## Abstract

Background and aims: Adherence to guidelines is associated with improved long-term outcomes in patients with chronic hepatitis B (CHB). We aimed to study the degree of adherence and determinants of non-adherence to management guidelines in a low endemic country. Methods: We reviewed the medical records of all CHB patients who visited our outpatient clinic in 2020. Adherence to guidelines was assessed based on predefined criteria based on the EASL guidance, and included the initiation of antiviral therapy when indicated, the optimal choice of antiviral therapy based on comorbidities, an assessment of HAV/HCV/HDV/HIV serostatus, renal function monitoring and enrolment in a HCC surveillance program if indicated. The adherence rates were compared across types of outpatient clinic (dedicated viral hepatitis clinic versus general hepatology clinic). Results: We enrolled 482 patients. Among the 276 patients with an indication for antiviral therapy, 268 (97.1%) received treatment. Among the patients with renal and/or bone disease, 26/29 (89.7%) received the optimal choice of antiviral agent. The assessment of HAV/HCV/HDV/HIV serostatus was performed in 86.1/91.7/94.4/78.4%. Among the 91 patients treated with tenofovir disoproxil, 57 (62.6%) underwent monitoring of renal function. Of the 241 patients with an indication for HCC surveillance, 212 (88.3%) were enrolled in a surveillance program. Clinics dedicated to viral hepatitis had superior adherence rates compared to general hepatology clinics (complete adherence rates 63.6% versus 37.2%, *p* < 0.001). Conclusions: Follow-up at a dedicated viral hepatitis clinic was associated with superior adherence to management guidelines.

## 1. Introduction

Chronic hepatitis B (CHB) is a global healthcare problem which currently affects approximately 248 million persons worldwide [1,2]. The prevalence of CHB varies widely across countries. In Western nations the prevalence of CHB is generally low (<2%), whereas the prevalence may be up to 5% in North Africa, 5–8% in parts of Asia and is estimated to be over 8% in sub-Saharan Africa [3]. In the Netherlands, the prevalence has been estimated at 0.34% [4]. Various studies have hinted that the uptake of recent recommendations with regard to the optimal choice of antiviral therapy and hepatocellular carcinoma (HCC) surveillance is suboptimal, leading to worse outcomes for patients [5,6,7,8]. This may be particularly relevant in countries with a low hepatitis B virus (HBV) prevalence, as expertise may vary across physicians. We therefore sought to investigate the degree of adherence to management guidelines in a low endemic country.

## 2. Methods

### 2.1. Study Design and Study Population

This study is part of an Initiative to Improve CARe for cUrrent and future patients with chronic hepatitis B in The Netherlands (ICARUS). For this study, all consecutive patients with HBV mono-infection (defined as HBsAg positivity for at least six months) who visited the outpatient clinic of the department of Gastroenterology and Hepatology of the Erasmus MC in 2020 were enrolled. The Erasmus MC, University Medical Center, is a large tertiary referral hospital located in the centre of Rotterdam, the second largest city in the Netherlands.

Patients with CHB are seen at either one of the dedicated viral hepatitis outpatient clinics or at one of several general hepatology outpatient clinics. Both the viral hepatitis and general hepatology clinics were overseen by experienced hepatologists. General hepatitis clinics care for patients with a variety of liver diseases, with viral hepatitis accounting for a minority of the patient population, whereas the viral hepatitis clinics cater exclusively to patients with viral hepatitis. Patients with viral hepatitis are preferably allocated to one of the viral hepatitis clinics. However, due to lack of capacity and/or patient preference, some patients cannot attend one of the viral hepatitis clinics and are, therefore, managed at general hepatology clinics. Allocation is thus unrelated to the severity of liver disease or phase of HBV infection.

### 2.2. Data Collection

For all eligible patients, patient charts were reviewed, and data were obtained regarding patient demographics, virology, stage of liver disease and relevant comorbidities. Diagnosis of cirrhosis was based on histology, ultrasound findings compatible with cirrhosis in the presence of signs of portal hypertension or a liver stiffness of ≥12.5 kPa (based on Fibroscan^®^, Paris, France).

### 2.3. Definition of Standard of Care

Standard of care was based upon the recommendations set forth in the European Association for the Study of the Liver (EASL) guideline [1]. Adherence to the guideline was assessed through ascertainment of compliance with several predefined criteria covering the whole spectrum of CHB care, including (1) treatment indications; (2) optimal choice of therapy in the presence of renal and/or bone disease; (3) assessment HAV, HCV, HDV and HIV serostatus; (4) monitoring of renal function during high-risk treatment; and (5) enrolment in HCC surveillance programs if indicated.

Patients meeting any of the following criteria were considered to be in need of antiviral therapy with nucleo(s)tide analogues: the presence of cirrhosis with detectable HBV DNA levels; ≥F2 liver fibrosis with HBV DNA > 2000 IU/mL and ALT > the upper limit of normal (ULN); HBV DNA > 20,000 IU/mL and ALT > 2× ULN, or serum HBV DNA > 10^7^ IU/mL (irrespective of fibrosis stage); positive family history for cirrhosis or HCC; presence of extra-hepatic symptoms; patients starting on high-risk immunosuppressive agents; and HBV DNA > 200,000 UI/mL in pregnant women.

The optimal choice of antiviral therapy in patients with renal and/or bone disease was defined as the use of tenofovir alafenamide (TAF) or entecavir (ETV) among patients with an indication for antiviral therapy.

The presence of (protection against) co-infections was studied by an assessment of the presence of IgG anti-HCV, IgG anti-HIV, IgG anti-HAV and IgG anti-HDV at least once prior to a patient’s visit to the outpatient clinic in 2020.

Adequate monitoring of renal function was defined as the regular (at least yearly) assessment of serum creatinine levels and serum phosphate levels in patients treated with tenofovir disoproxil (TDF).

Indications for HCC surveillance comprised either a positive family history of HCC, the presence of liver cirrhosis or ethnicity (Sub-Saharan patients aged ≥20 years, Asian male patients aged ≥40 years and Asian female patients aged ≥50 years). Adequate HCC surveillance was defined conservatively as the presence of at least three imaging studies in the previous two years.

### 2.4. Outcomes and Statistical Analysis

The primary outcome of this study was the proportion of patients not managed according to the guidelines, as assessed using the individual indicators described above. We also calculated a composite measure of complete adherence, which was based on adherence to all individual components. The adherence rates were assessed in the overall population and stratified according to the type of outpatient clinic (dedicated viral hepatitis clinic versus general hepatology clinic).

Descriptive data were described as numbers (with percentages), medians (with interquartile range; IQR) and means (±standard deviation; SD). The association between the type of outpatient clinic and adherence was explored using a chi square test. Differences were considered statistically significant when *p* < 0.05. For the statistical data analysis, IBM SPSS for Windows version 25.0 (SPSS Inc., Chicago, IL, USA), was used.

### 2.5. Ethical Considerations

The study was conducted in accordance with the guidelines of the declaration of Helsinki and the principles of Good Clinical Practice. The study was also reviewed by the Institutional Review Board of the Erasmus MC (MEC-2020-0823).

## 3. Results

### 3.1. Patient Characteristics

We enrolled 482 patients. The patient characteristics are shown in Table 1. The mean age was 49 years (±14) and 54.4% were male. The most common ethnicities were Asian (38.2%) and North African/Middle Eastern (26.1%; Table 1). Liver cirrhosis was present in 12.0% of the patients.

### 3.2. Adherence Rates in the Overall Population

Overall, complete adherence to all assessed components was observed in 254 (52.7%) patients.

In total, 276 (57.3%) patients had an indication for antiviral therapy. The most common indication was HBV DNA > 20,000 IU/mL and ALT > 2× ULN (116/276, 42.0%). Among the patients with an indication for antiviral therapy, 268 (97.1%) received antiviral therapy (Table 2).

Among the 29 patients with renal and/or bone disease, 26 (89.7%) were treated with TAF or ETV (Table 2).

IgG anti-HAV, anti-HCV and anti-HDV were assessed in the majority of patients (>86%), whereas anti-HIV was assessed in 378 (78.4%; Table 2).

In total, 91 patients were treated with TDF. None of the patients were treated with ADV in 2020. Creatinine and phosphate level measurements were regularly performed in 57/91 (62.6%) patients (Table 2).

In total, 241 (50.0%) had at least one HCC risk factor and, therefore, had an indication for HCC surveillance. Among these, 212 (88.3%) received adequate surveillance (Table 2). Among the 58 patients with cirrhosis, 52 (89.7%) underwent adequate surveillance.

### 3.3. Dedicated Viral Hepatitis Clinics Have Superior Adherence Rates

Among the 283 patients treated at a dedicated viral hepatitis clinic, complete adherence to all indicators was observed in 180 patients (63.6%), compared to 74/199 patients (37.2%) that were treated at a general hepatology clinic (*p* < 0.001). The findings were consistent across the individual indicators, such as renal function monitoring (68.9 vs. 34.6%, *p* < 0.001); assessment of HAV (94.0 vs. 74.9%, *p* < 0.001); HCV (94.0 vs. 88.4%, *p* = 0.030); HDV (96.8 vs. 91.0%, *p* = 0.006); and HIV (84.5 vs. 69.8%, *p* < 0.001) serostatus. Adequate HCC surveillance rates were higher among dedicated viral hepatitis clinics (92.4 vs. 82.3%, *p* = 0.016), particularly among patients without liver cirrhosis (94.1 vs. 76.9%, *p* < 0.001). Adherence to guidelines for the initiation of antiviral therapy was high regardless of clinic type (*p* = 0.159). The findings were consistent after adjustment for patient age, sex and ethnicity.

## 4. Discussion

Adherence to guidelines is of paramount importance for improving patient care. In the current study conducted in a large tertiary care hospital in a low endemic country, the majority of patients received optimal care based on EASL guideline recommendations. However, a significant number of patients remained untested for viral co-infections, did not receive the optimal antiviral agent based on comorbidities and, perhaps most importantly, did not receive adequate HCC surveillance. A major risk factor for non-adherence to guideline recommendations was management at a non-viral hepatitis specialised liver clinic, suggesting that centralising care for CHB patients may be critical in optimising clinical management.

CHB is a complex disease with a heterogeneous natural history. Treatment indications are established on multiple factors based on biochemistry, virology and stage of liver disease, as well as patient factors including a family history of liver-related complications. When deciding on initiating antiviral therapy, the optimal choice of antiviral agent is not just based on virological factors but should also take into consideration the presence of comorbidities such as renal or bone diseases. During treatment, follow-up for treatment related complications is mandatory in some, but not all, agents. We observed that the majority of patients with a treatment indication received antiviral therapy, although ~3% of patients with obvious treatment indications remained untreated. Even though the specific reason for under-treatment are difficult to ascertain from our retrospective study (patient-related or physician-related), this is a missed opportunity, as antiviral therapy may improve liver histology and reduce the risk of HCC and the development of cirrhosis [9].

Recent studies indicate that treatment with TDF may be a risk factor for impaired proximal tubular reabsorption, which is known as Fanconi syndrome [10]. The monitoring of renal function and serum phosphate levels is, therefore, advised in both the guidelines and Summary of Product Characteristics (SMPCs). However, we observed that 63% of the patients treated with TDF were not monitored for creatinine and phosphate levels, suggesting that this potentially devastating complication requires more attention.

In addition, we observed a suboptimal screening rate for co-infections, especially the measurement of anti-HIV. HIV–HBV co-infection accelerates the progression of liver related complications such as liver cirrhosis or HCC compared to patients with an HBV mono-infection. Moreover, among patients with an undiagnosed HIV co-infection, treatment with a single antiviral agent could induce drug resistance [11]. Therefore, the identification of patients with a co-infection is of clinical importance.

A final important aspect of CHB management is identifying patients at a high risk of developing HCC. Aside from the presence of cirrhosis as an established risk factor, various other subgroups have also been identified as high risk and considered eligible for enrolment in HCC surveillance programs, such as combinations of ethnicity, age and family history. It is, therefore, not surprising that the management of CHB is challenging for most physicians, especially in low-endemic countries where physicians may care for limited numbers of CHB patients and so build limited experience. In the current study, 50% of patients were potentially eligible for HCC surveillance. Only 88% of these patients underwent adequate HCC surveillance during the study timeframe. Notably, HCC surveillance was adequately performed among 82% of the patients who were managed at general hepatology clinics, compared to 92% in patients seen at a dedicated viral hepatitis clinic (*p* = 0.016). The suboptimal HCC surveillance rates are in line with previous data [12] and are unfortunate, as HCC surveillance has been associated with improved outcomes [5].

The findings reported here corroborate those from a previous study [13] and are in line with reports from other fields [14,15,16]. Furthermore, we have recently published a study showing that general practitioners generally do not provide adequate follow-up to patients with viral hepatitis, despite the availability of a specific guideline [17], further underscoring the importance of centralizing care in CHB. Additional interventions that could potentially improve adherence are focused on training sessions and/or the implementation of tools in the electronic medical records that support standardised care, for example, through reflex testing for co-infections in patients with viral hepatitis.

The strengths of this study include a large cohort of CHB patients that visited a large academic hospital in 2020. Several limitations of this study should be considered. First, the retrospective design of the study utilized data from patient charts, which may not always contain complete information on potential reasons for deviating from the guidelines. Secondly, this study has been conducted in a tertiary centre with high expertise for liver-related care. Whether our findings could be extrapolated to other (non-)academic hospitals, or other low endemic countries, warrants further exploration.

In conclusion, the majority of CHB patients in our hospital were monitored and treated according to the management guidelines. Follow-up at a dedicated viral hepatitis outpatient clinic was associated with superior adherence rates.

## Figures and Tables

**Table 1:** Patient characteristics.

	*n* = 482
**Age** (years; mean, SD)	49 (±14)
**Sex** (male; *n*, %)	262 (54.4)
**Ethnicity** (*n*, %)	
Caucasian, White	78 (16.2)
Asian	184 (38.2)
Black, Sub-Saharan	66 (13.7)
Black, non-Sub-Saharan	24 (5.0)
North-African or Middle Eastern countries	126 (26.1)
Hispanic	4 (0.8)
**Comorbidities** (*n*, %)	
Osteoporosis	5 (1.0)
Renal dysfunction ^α^	32/395 (8.1)
**Liver stiffness** (kPa; median, IQR)	6.1 (4.7–8.3)
**Liver cirrhosis** ^β^ (*n*, %)	58 (12.0)

*^α^ Renal dysfunction was defined as an eGFR under 60 mL/min/1.73 m^2^. ^β^ Diagnosis of cirrhosis was based on histology, ultrasound findings compatible with cirrhosis in the presence of signs of portal hypertension or a liver stiffness measurement of ≥12.5 kPa.*

**Table 2:** Adherence of clinical guideline [1].

*n*, %	Adequate Adherence Guideline	Inadequate Adherence Guideline	Total
**Indication to start therapy**	268 (97.1)	8 (2.9)	276
- Cirrhosis with detectable HBV DNA levels (>20 IU/mL)	56 (100)	0 (0)	56 ^β^
- HBV DNA > 2000 IU/mL with ALT > ULN and at least F2 fibrosis	35 (97.2)	1 (2.8)	36
- HBV DNA > 20,000 IU/mL with ALT > 2× ULN	111 (95.7)	5 (4.3)	116
- HBV DNA > 107 IU/mL	24 (96.0)	1 (4.0)	25
- Positive family history for cirrhosis or HCC	15 (100)	0 (0)	15
- Presence of extra-hepatic symptoms	0 (0)	0 (0)	0
- Starting immunosuppressive agents	25 (96.2)	1 (3.8)	26
- HBV DNA > 200,000 UI/mL in pregnant women	2 (100.0)	0 (0)	2
**Optimal choice of antiviral**			
- Use of TAF or ETV in patients with renal or bone disease	26 (89.7)	3 (10.3)	29
**Assessment of serostatus ^∑^**			482
- anti-HAV (IgG)	415 (86.1)	67 (13.9)	
- anti-HCV (IgG)	442 (91.7)	40 (8.5)	
- anti-HDV (IgG)	455 (94.4)	27 (5.6)	
- anti-HIV (IgG)	378 (78.4)	104 (21.6)	
**Monitoring creatinine and phosphate levels among patients treated with TDF ^¥^**	57 (62.6%)	34 (37.4)	91
**HCC surveillance among patients with an indication ^π^**	212 (88.3)	28 (11.7)	241

*^β^ The total cohort included 58 patients with liver cirrhosis. However, two patients were excluded from the analysis as the viral load was undetectable. ^∑^ Assessment of the presence of IgG anti-HCV, IgG anti-HIV, IgG anti-HAV and IgG anti-HDV at least once during follow-up; ^¥^ Adequate monitoring was defined as the quantification of serum creatinine and phosphate levels at least once a year among patients treated with ADV, TDF or TAF. ^π^ Enrolment in a HCC surveillance program if indicated based on family history of HCC, presence of liver cirrhosis and ethnicity (Sub-Saharan patients aged ≥20 years, Asian male patients aged ≥40 years and Asian female patients aged ≥50 years). Adequate HCC surveillance was defined conservatively as at least three ultrasounds (or one MRI) performances in the previous two years. Abbreviations: ALT, alanine aminotransferase; ETV, entecavir; HAV, hepatitis A virus; HCC, hepatocellular carcinoma; HCV, hepatitis C virus; HDV, hepatitis delta virus; HIV, human immunodeficiency virus; IU/mL, international units/millilitre; TAF, tenofovir alafenamide; TDF, tenofovir disoproxil; ULN, upper limit of normal.*

## Data Availability

Data not available.

## Abbreviations

ALT	alanine aminotransferase
CHB	chronic hepatitis B
EASL	European Association for the Study of the Liver
ETV	entecavir
HAV	hepatitis A virus
HBV	hepatitis B virus
HBsAg	hepatitis B surface antigen
HCC	hepatocellular carcinoma
HCV	hepatitis C virus
HDV	hepatitis delta virus
HIV	human immunodeficiency virus
IU/mL	international units/millilitre
kPa	kilopascal
TAF	tenofovir alafenamide
TDF	tenofovir disoproxil
SMPCs	Summary of Product Characteristics
ULN	upper limit of normal
U/mL	units/millilitre
